# Fabrication of a uniform chromate conversion coating on Zn alloy for improved corrosion resistance in humid environments

**DOI:** 10.1038/s41598-023-41629-w

**Published:** 2023-08-31

**Authors:** Seonghye Ha, Jakyung Eun, Changhoon Choi, Soohyoun Cho, Sangmin Jeon

**Affiliations:** 1 Department of Chemical Engineering, Pohang University of Science and Technology (POSTECH), 77 Cheongam-Ro, Pohang, Gyeongbuk Republic of Korea; 2 POSCO, 8 Pokposarang-Gil, Gwangyang-Si, Jeollanam-Do Republic of Korea

**Keywords:** Engineering, Nanoscience and technology

## Abstract

We developed a facile method to produce a uniform chromate conversion (CC) coating on zinc alloy-plated steel substrates (ZS). When an acidic CC solution is applied to ZS (C-ZS), zinc is dissolved and chromium ions are reduced to form a chromate coating. In localized areas where zinc is excessively dissolved, zinc hydroxide particles are formed, which hinders the formation of a uniform chromate film, leaving the areas vulnerable to further corrosion (i.e., the formation of dark spots) when exposed to high humidity conditions. To suppress the excessive dissolution of zinc, the ZS surface was pretreated with thiolated polyethylene oxide to form a hydrophilic self-assembled monolayer. A more uniform protective CC coating was obtained on the pretreated ZS, resulting in superior corrosion resistance under high humidity conditions.

## Introduction

Zinc alloy-plated steel (ZS) is widely used in various industrial applications because of its excellent resistance to corrosion^1, 2^. To improve the corrosion resistance of ZS further, an acidic chromate conversion (CC) coating is often used as a post-treatment process^3, 4^. When an acidic CC solution is applied to ZS, the anodic dissolution of zinc leads to the cathodic precipitation of chromium oxide, which acts as a barrier to protect the underlying zinc alloy from corrosive environments^1, 5, 6^.

While CC coatings have proven effective in preventing general corrosion, studies have reported the appearance of small dark spots on CC-coated ZS (C-ZS) under high humidity conditions^7, 8^. These dark spots are caused by zinc corrosion products, such as zinc oxide and zinc hydroxides. Although zinc oxide and zinc hydroxides are usually white in color, they can appear dark due to variations in the optical properties of the corrosion products and the underlying zinc^7^. Once dark spots appear, they can induce the peeling of an applied organic film, such as paint, and can lead to the development of larger white corrosion products^8, 9^. Therefore, the development of a method to suppress dark spot formation during the CC coating process is needed.

In this study, it was revealed that the appearance of dark spots on the C-ZS surface was initiated by the localized damage inflicted on ZS during the application of the acidic CC coating. The regions that were left without adequate coverage were prone to developing into dark spots when exposed to high humidity. To mitigate surface damage, the ZS surface was pretreated with a thiolated polyethylene oxide (PEO-SH) self-assembled monolayer (SAM) before applying the CC coating. While hydrophobic SAMs have been employed to protect metal substrates from aggressive water and acid molecules^10–12^, to the best of our knowledge, this is the first attempt to utilize a hydrophilic SAM to achieve a uniform CC coating on ZS. The surface of C-ZS treated with an interlayer of PEO-SH (CP-ZS) showed minimal damage and effectively prevented the formation of dark spots during CC coating treatment. The resulting CP-ZS demonstrated superior corrosion resistance to C-ZS under high humidity environments.

## Methods

### Materials

Hot-dipped Zn alloy-plated steel was obtained from POSCO (Korea). The zinc alloy layer comprises 97% Zn, 1.5% Mg, and 1.5% Al. Cr(NO_3_)_3_ and ethanol (absolute) were purchased from UNICOH (Korea) and Sigma-Aldrich (St. Louis, MO), respectively. Due to the carcinogenic nature of hexavalent chromium, we utilized trivalent chromium for the conversion coating process. Thiolated polyethylene oxide (PEO-SH, Mw: 356 g/mol) was procured from Polypure (Oslo, Norway). Nitric acid (60%) and methyl ethyl ketone were obtained from SAMCHUN (Korea) and DAE JUNG (Korea), respectively. Deionized (DI) water (18.3 MΩ·cm) was obtained using a reverse osmosis water system (Human Corporation, Korea).

### Preparation of C-ZS and CP-ZS

A 3 wt.% solution of Cr(NO_3_)_3_ in 7:3 (v:v) DI water and ethanol was prepared as the CC coating solution. Following typical commercial processes^13, 14^, the pH of the CC coating solution as adjusted to 1.6 using 20 wt.% nitric acid. Ethanol was employed to enhance the wettability of the solution on ZS. It is worth noting that the use of volatile organic compounds in industrial production is limited to a low amount. The PEO-thiol solution was prepared by dissolving PEO-SH in absolute ethanol to a final concentration of 10 mM. ZS samples were cut into 3.7 × 3.7 cm squares, degreased in methyl ethyl ketone, and then sonicated in ethanol for 3 min. Thereafter, ZS squares were immersed in the PEO-thiol solution for 24 h to obtain P-ZS. The CC solution was then applied to ZS and P-ZS using a bar coater to create C-ZS and CP-ZS, respectively. C-ZS and CP-ZS samples were then dried in a 100 °C oven at a peak metal temperature of 60 °C.

### Analyses of dark spots formed on ZS, C-ZS, and CP-ZS

To compare the formation of dark spots on ZS, C-ZS, and CP-ZS, each sample was exposed to a temperature and humidity chamber (MEAN science) set to 40 °C and 95% RH for 72 h. The surface and cross-section of the CC coatings were then examined using optical microscopy (OM, Leica model DVM6a) and scanning electron microscopy equipped with energy dispersive X-ray spectroscopy (SEM-EDS, JEOL model JSM 7800F). Cross-section pieces were polished using an ion beam cross-section polisher (Leica model EM TIC 3X) and a 60 nm thick Pt layer was sputtered on to distinguish the CC coating from the layer deposited during ion beam milling.

## Results and discussion

**Figure 1:**
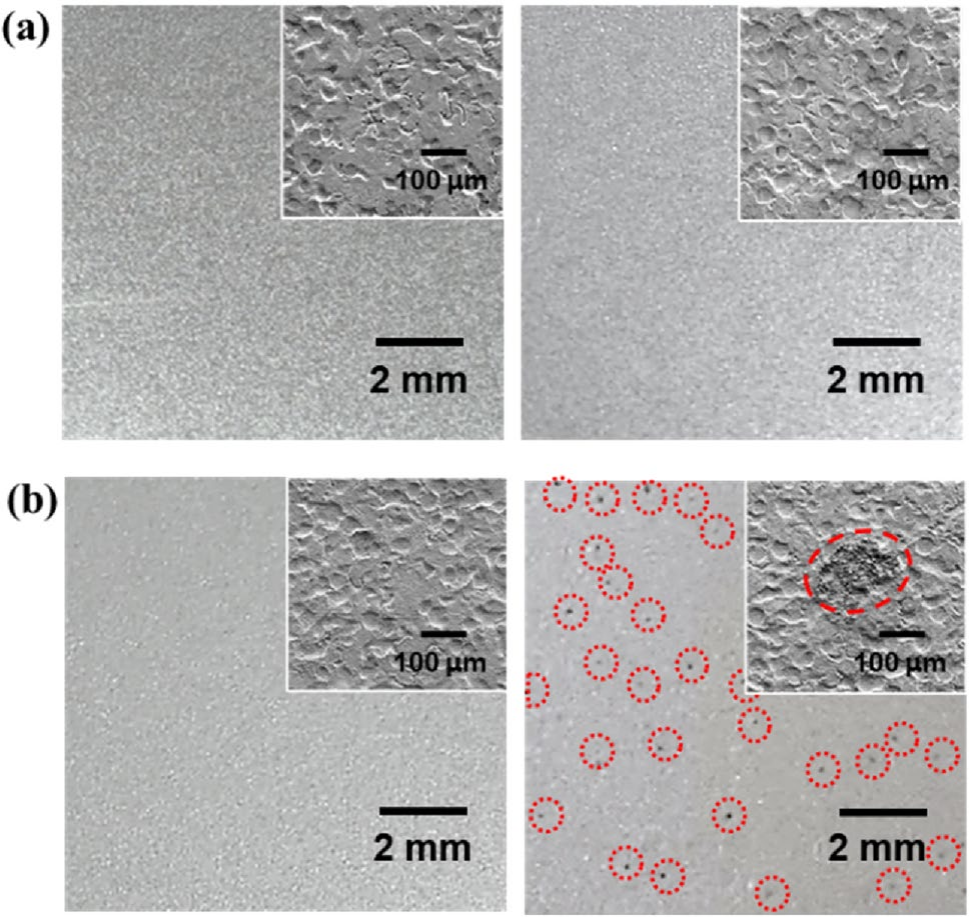
OM images of (**a**) ZS and (**b**) C-ZS before (left panel) and after (right panel) humidity treatment (95% RH at 40 ℃ for 72 h). The red dotted circles in (**b**, right panel) highlight the position of black spots. Insets show the SEM image of each surface.

Figure 1a,b show OM images of ZS and C-ZS before and after treatment in the humidity chamber (95% RH at 40℃ for 72 h). The inset in each image displays a surface SEM image and the surface roughness could be attributed to the skin-pass process^15, 16^. The surface morphology of C-ZS was similar to that of ZS, indicating that the very thin (~ 100 nm) CC coating did not substantially alter the surface morphology. After exposure to high humidity, only negligible morphological changes were observed on the ZS sample; however, dark spots appeared on the C-ZS sample, indicating that the dark spots were associated with the CC coating process.

**Figure 2:**
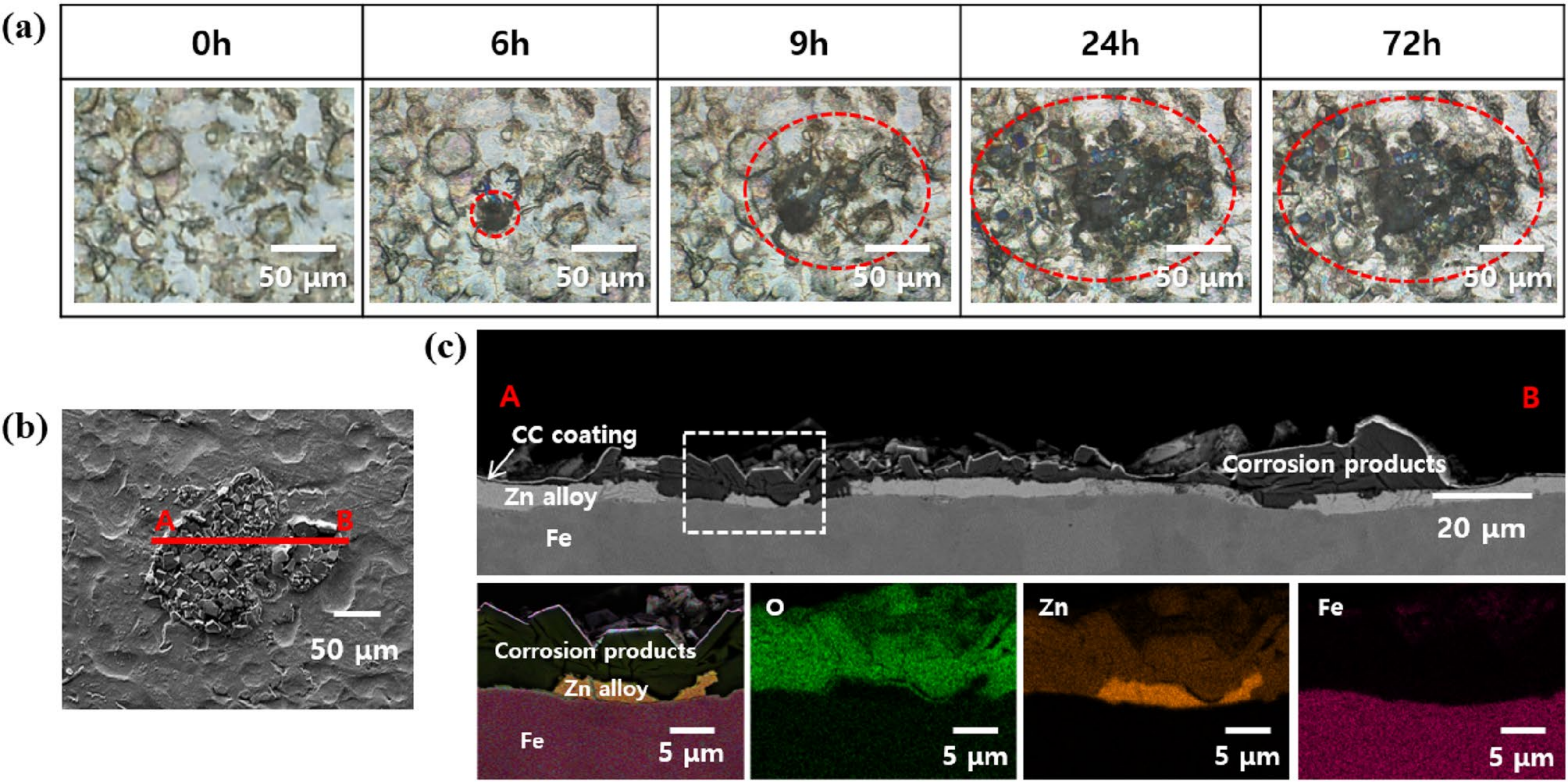
(**a**) Time-lapse OM images of dark spot formation on C-ZS under humidity treatment. The red dotted circle highlights the location of the dark spot. (**b**) Top-view SEM image of the dark spot on C-ZS after exposure to humidity for 72 h. (**c**) Cross-sectional SEM image of C-ZS after exposure to humidity for 72 h (upper panel). The red A and B correspond to the same A and B positions in (**b**). The enlarged SEM image of the region enclosed by the white dotted box, along with the associated EDS mappings, are displayed in the lower panel.

Figure 2a shows the time-lapse OM images of the same location on C-ZS during the humidity treatment. A dark spot with a diameter of ~ 20 µm formed after 6 h of exposure. After 72 h, this spot expanded to ~ 100 µm. This suggests that the dark spot resulted from localized corrosion under high humidity conditions. Figure 2b shows the top-view SEM image of the dark spot after humidity treatment for 72 h. The dark spot consisted of particles ~ 15 µm in size. A cross-sectional SEM image of the dark spot is shown in the upper panel of Fig. 2c. Note that the red A and B in Fig. 2b,c correspond to the same location. Particles were observed both on the surface of the zinc alloy layer and the steel substrate beneath, demonstrating the depth of corrosion. The enlarged SEM image of the region enclosed by the white dotted box, along with the associated EDS mappings are shown in the lower panels of c. The observed particles were primarily composed of oxygen and zinc, indicating that the dark spot is composed of zinc-containing oxides or hydroxides, corrosion products of zinc in a humid environment.

**Figure 3:**
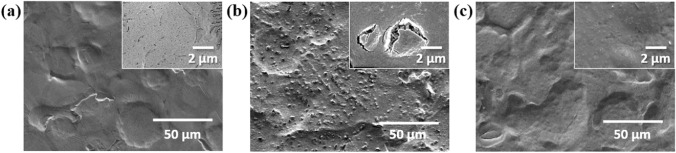
SEM images of (**a**) ZS, (**b**) C-ZS, and (**c**) CP-ZS before exposure to high humidity treatment. Insets of (**a**)–(**c**) show the magnified SEM images.

If a uniform and dense CC coating is applied to ZS, it should effectively protect the underlying zinc layer from corrosion. Therefore, the formation of dark spots on C-ZS is believed to be caused by surface damage during the CC coating under acidic conditions. To test this hypothesis, a hydrophilic PEO-SH layer was applied to the surface of ZS before the CC coating was applied. After coating with PEO-SH, the water contact angles of ZS changed from 79° to 43°, indicating the formation of a hydrophilic PEO layer on P-ZS (Fig. S1 in the Supplementary Information). Figure 3a–c show the surface morphologies of ZS, C-ZS, and CP-ZS, respectively, before high humidity treatment. The differences in morphology and structure can also be observed in their cross-sectional SEM images (Fig. S2 in the Supplementary Information). ZS exhibits a smooth plateau–valley surface, resulting from the skin-pass process, with no visibly damaged areas. In contrast, the surface of C-ZS appears irregular and is characterized by the presence of numerous protrusions measuring 1–2 μm in size. Cross-sectional SEM and EDS analysis revealed that these protrusions were zinc oxides or hydroxides, indicating localized corrosion of the zinc alloy layer during acidic CC coating treatment (Fig. S3 in the Supplementary Information). The magnified image in the inset of Fig. 3b shows that the CC coating layer on the protrusion was broken. Therefore, C-ZS was not uniformly coated, and the protrusions are gaps in the coating that make C-ZS susceptible to further corrosion when exposed to high humidity conditions. The surface morphology of CP-ZS was similar to that of ZS, with no visibly damaged areas, indicating that the self-assembled PEO layer effectively protects the surface from damage by aggressive acid and water molecules^10^.

**Figure 4:**
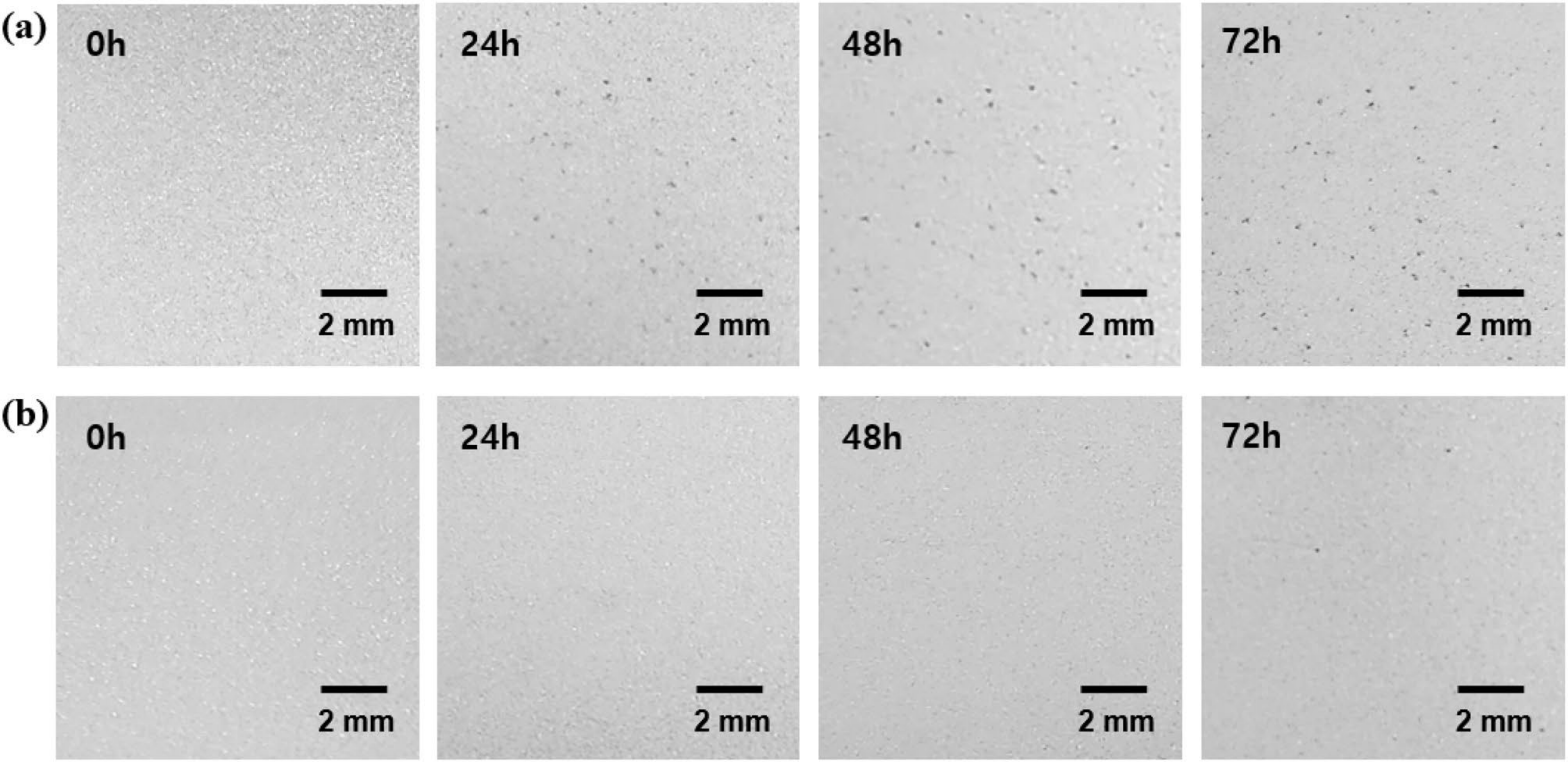
Time-lapse OM images of dark spots formed on (**a**) C-ZS and (**b**) CP-ZS after humidity treatment (95% RH at 40 °C) for 0 h, 24 h, 48 h, and 72 h.

The effectiveness of the self-assembled PEO layer in preventing the formation of dark spots on CP-ZS was evaluated by subjecting CP-ZS to high humidity conditions. Figure 4a,b show time-lapse OM images of dark spots formed on C-ZS and CP-ZS when exposed to 95% RH at 40 °C. Approximately 50 dark spots appeared on C-ZS after 24 h of exposure, and this number increased to approximately 58 after 72 h. In contrast, no dark spots were observed for 48 h on CP-ZS, and only 4 dark spots appeared after 72 h, confirming that the self-assembled PEO layer was effective in protecting the surface from localized corrosion. A control experiment was conducted to investigate whether the PEO-SH coating protects the anodic dissolution of ZS. After treating ZS and P-ZS with an HNO_3_ in ethanol solution, using the same pH (1.6) as the CC coating solution, a significant increase in oxygen content was observed on ZS, while only a negligible change in oxygen content was seen on P-ZS (Fig. S4 in the Supplementary Information), confirming the effective suppression of zinc dissolution by the PEO-SH coating.

## Conclusion

In this study, we developed an effective method to minimize surface damage of Zn alloy-plated steel during the CC coating process. Employing PEO-SH as a surface pretreatment before the CC coating process resulted in a uniform CC coating layer with improved corrosion resistance under high humidity conditions (95% relative humidity at 40 °C). Utilization of a PEO-SH surface modifier in the CC coating process effectively reduced the formation of dark spots on ZS, indicative of corrosion, when exposed to high humidity conditions. As PEO-SH spontaneously forms on zinc surfaces via covalent bonding under ambient conditions, the developed method has the potential for improving the corrosion resistance of ZS in a wide range of applications.

### Supplementary Information

Supplementary Figures.

## Data Availability

The datasets used and/or analysed during the current study available from the corresponding author on reasonable request.
