# Supplementary material for: Fabrication of a uniform chromate conversion coating on Zn alloy for improved corrosion resistance in humid environments
Source: Sci Rep. 2023 Aug 31;13:14311. doi: 10.1038/s41598-023-41629-w (PMC10471749; doi:10.1038/s41598-023-41629-w)
Supplement: Supplementary file 1 — Supplementary Figures. [file 41598_2023_41629_MOESM1_ESM.docx]

Supplementary information

**Fabrication of a Uniform Chromate Conversion Coating on Zn Alloy for Improved Corrosion Resistance in Humid Environments**

Seonghye Ha^1^, Jakyung Eun^1^, Changhoon Choi^2^, Soohyoun Cho^2^, and Sangmin Jeon^1^*

^1^ Department of Chemical Engineering, Pohang University of Science and Technology (POSTECH), 77 Cheongam-Ro, Pohang, Gyeongbuk, Republic of Korea

^2^ POSCO, 8 Pokposarang-gil, Gwangyang-si, Jeollanam-do, Republic of Korea


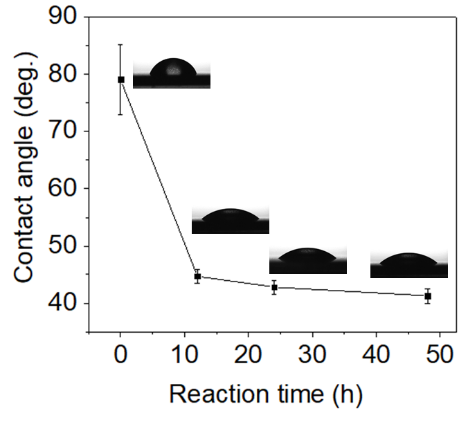


**Figure S1.** Changes in water contact angles of ZS as a function of 10 mM PEO-SH treatment time. The decreasing WCA with treatment time indicates an increase in surface hydrophilicity, suggesting enhanced PEO-SH adsorption.


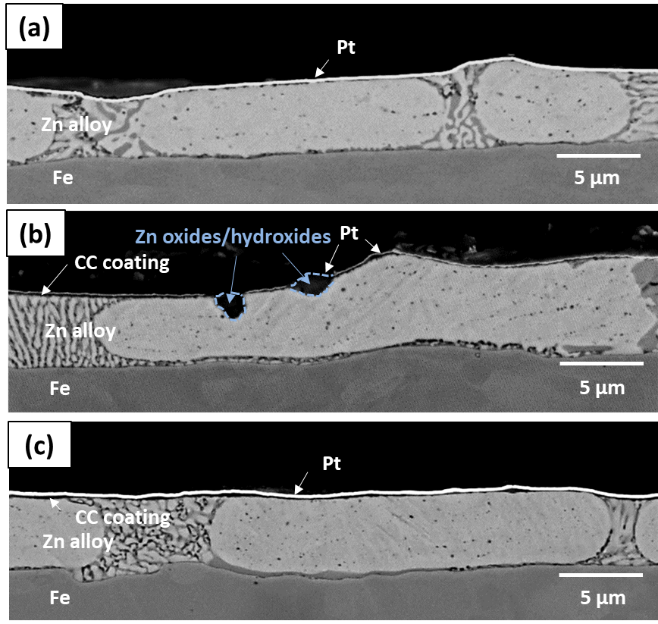


**Figure S2.** Cross-sectional SEM image of (a) ZS, (b) C-ZS, and (c) CP-ZS. The white lines at the uppermost part correspond to the platinum layer, which was used to protect the surface during cutting, and the black lines immediately below the platinum layer correspond to the CC layer. In contrast, in ZS, the Pt layer is directly on the Zn alloy layer, confirming the absence of the CC layer.

**
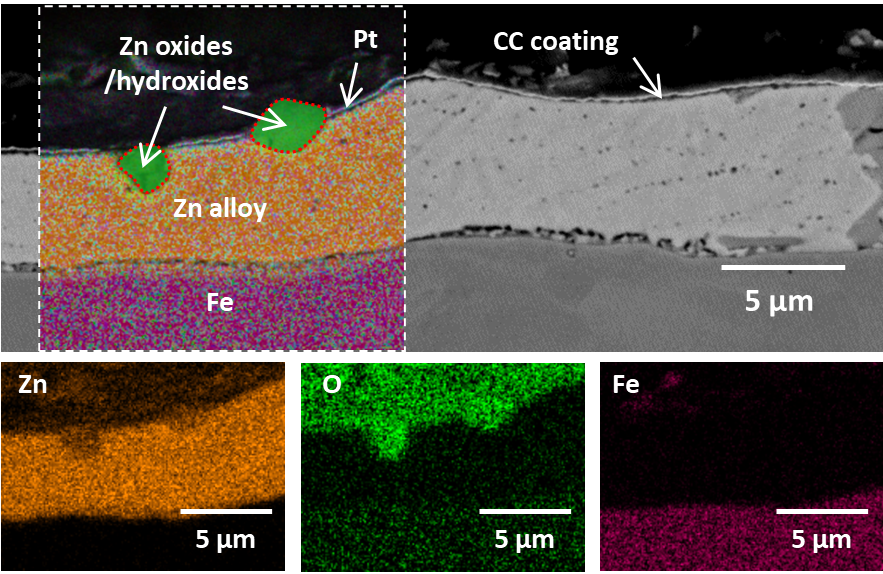
**

**Figure S3.** Cross-sectional SEM image of C-ZS after CC coating. The magnified SEM image and EDS mapping in the white dotted box of the upper panel are shown in the lower panel. The small amount of Fe observed in the cross-sectional EDS mapping image originates from contaminants introduced during ion beam milling for sample preparation.


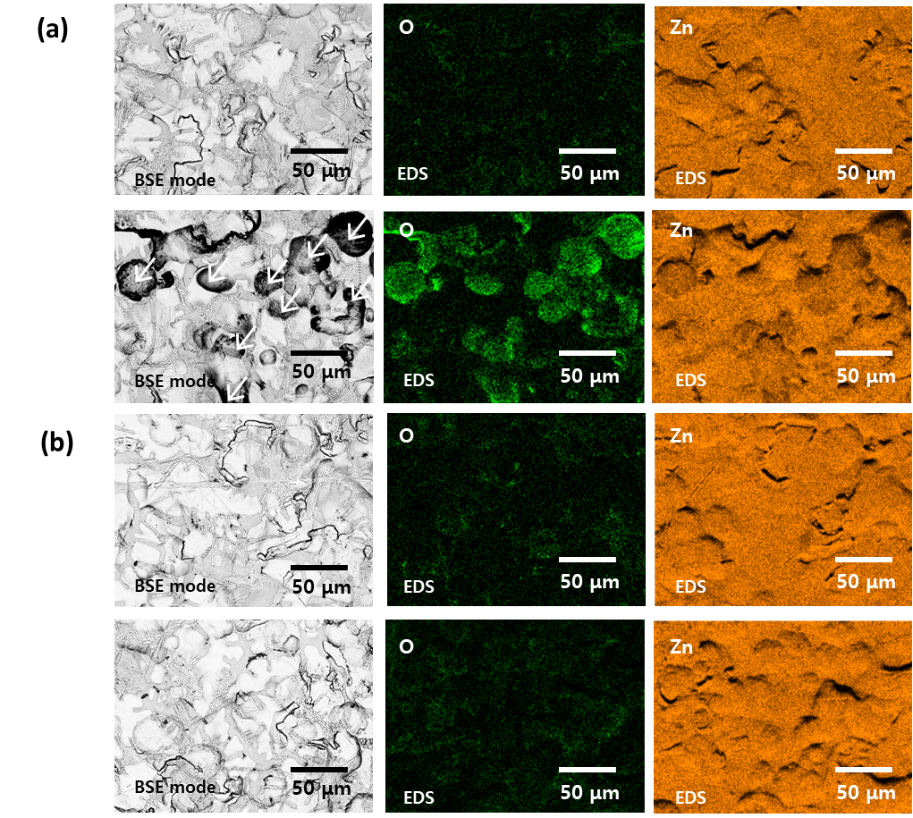


**Figure S4.** SEM images (a) ZS and (b) P-ZS before (upper panel) and after (lower) HNO_3_ treatment. Each surface was coated using an HNO_3_ in ethanol solution with the same pH (1.6) as the CC coating solution. After the HNO_3_ treatment, a substantial increase in oxygen content was observed on ZS, while only a negligible change in oxygen content was seen on P-ZS, indicating that the PEO-SH coating effectively suppressed the dissolution of zinc.
